# Melatonin Ameliorates Desiccation Stress‐Induced Ocular Inflammation in an In Vitro Model by Activating the Nrf2 Pathway

**DOI:** 10.1111/jcmm.70879

**Published:** 2025-10-16

**Authors:** Hye‐Sun Lim, Jinheung Park, Woong‐Joo Whang, Wan Seok Kang, Sunoh Kim, Young‐Sik Yoo, Gunhyuk Park

**Affiliations:** ^1^ Herbal Medicine Resources Research Center Korea Institute of Oriental Medicine Naju‐si Jeollanam‐do Republic of Korea; ^2^ St. Mary‘s JIN Eye Center Seoul Republic of Korea; ^3^ Mediverse Co. Ltd. Seoul Republic of Korea; ^4^ Department of Ophthalmology, College of Medicine Yeouido St. Mary‘s Hospital, The Catholic University of Korea Seoul Republic of Korea; ^5^ Central R&D Center, B&Tech Co., Ltd. Naju Republic of Korea; ^6^ Department of Ophthalmology, College of Medicine Uijeongbu St. Mary‘s Hospital, The Catholic University of Korea Seoul Republic of Korea; ^7^ IU Eye Clinic Seoul Republic of Korea

**Keywords:** anti‐inflammation, desiccation stress, dry eye, melatonin, Nrf2

## Abstract

Dry eye disease (DED) is a multifactorial ocular disorder marked by tear film instability, oxidative stress, and inflammation. Although melatonin is known to exert antioxidant and anti‐inflammatory effects, its precise role in regulating desiccation‐induced stress responses at the ocular surface has not been well defined. In particular, the involvement of the Nrf2 signalling pathway in melatonin‐mediated protection against corneal epithelial inflammation under tear film instability remains unexplored. In this study, we evaluated the therapeutic potential of melatonin in a human corneal epithelial cell (HCEC) model mimicking desiccation stress. Melatonin treatment significantly reduced reactive oxygen species (ROS) levels and upregulated antioxidant enzymes, including catalase (CAT) and heme oxygenase‐1 (HO‐1), via Nrf2 pathway activation. These effects were attenuated by Nrf2 knockdown using siRNA, confirming pathway specificity. Furthermore, melatonin markedly suppressed pro‐inflammatory cytokine production, suggesting a dual mechanism of action involving both redox regulation and immune modulation. Our findings provide novel insight into the Nrf2‐dependent effects of melatonin on ocular surface protection under desiccation conditions. These results suggest melatonin as a promising candidate for the treatment of DED through simultaneous modulation of oxidative and inflammatory pathways specific to tear film instability.

## 1 Introduction

Chronic dry eye disease (DED) is an ocular surface disorder caused by tear deficiency, imbalanced tear composition, or altered tear osmolarity [1]. Its aetiology includes hormonal changes, environmental exposure, aging, autoimmune diseases, and refractive surgery [1, 2]. The prevalence of DED has increased owing to increased screen time [2]. Approximately 33% of elderly individuals (65+ years) have DED, representing a significant public health burden [3]. The growing prevalence of DED has led to rising healthcare costs, making DED a medical and economic concern [3].

DED treatments include artificial tears for lubrication, mucin secretagogues, such as diquafosol, and immunosuppressants, such as cyclosporine [1]. However, these therapies have limitations, including ocular irritation, surface toxicity, and poor patient adherence, owing to their requirement for frequent administration [2, 4]. Additionally, preservatives, such as benzalkonium chloride, can worsen ocular damage [2, 4]. Thus, alternative therapeutic strategies are required. One promising approach involves the regulation of oxidative stress, as impaired antioxidant defence exacerbates inflammation and disease progression.

Nuclear factor erythroid 2‐related factor 2 (Nrf2) is a key transcription factor that regulates antioxidant defence by promoting genes encoding catalase (CAT), NAD(P)H: quinone oxidoreductase 1 (NQO1), and heme oxygenase 1 (HO‐1) [5]. Nrf2 activation reduces oxidative stress and inflammation, highlighting its therapeutic potential in DED [6, 7, 8, 9]. Enhanced expression of antioxidant‐related genes may protect against oxidative damage and suppress ocular inflammation, making Nrf2 a promising therapeutic target [9].

Melatonin (N‐acetyl‐5‐methoxytryptamine) is an endogenous hormone primarily produced in response to darkness [10]. It regulates sleep, circadian rhythms, immune responses, and reproduction and exhibits strong antioxidant and anti‐inflammatory properties [11, 12]. Due to its ability to scavenge ROS and suppress pro‐inflammatory cytokines, melatonin has emerged as a potential therapeutic agent for various diseases [12].

Although melatonin has been studied in systemic and retinal models of oxidative damage, its protective role in desiccation‐induced ocular surface inflammation, particularly via Nrf2 activation in corneal epithelial cells, remains uncharacterised. This study evaluated the potential of melatonin as a novel treatment for DED by investigating its effects on Nrf2‐mediated antioxidant defence mechanisms and ocular surface protection.

## 2 Materials and Methods

### 2.1 Cell Culture

Human corneal epithelial cells (HCECs) were cultured under standard conditions (37°C, 5% CO_2_) in bronchial epithelial growth medium supplemented with necessary antibiotics and growth factors. Cells were subcultured using trypsin–EDTA once they reached approximately 80%–90% confluence and were routinely passaged every 3–4 days.

### 2.2 Induction of Desiccation Stress

To model ocular surface desiccation, HCECs were seeded onto transwell membranes and allowed to adhere overnight. The apical medium was removed to simulate dry conditions, while the basolateral chamber was replenished with fresh medium containing melatonin at specified concentrations. Cells were exposed to these conditions for 24 h prior to further analysis. The desiccation protocol was optimised based on a time‐course analysis of Nrf2 DNA‐binding activity (0–24 h), as shown in Figure S1. On the basis of these results, Nrf2 activation was primarily evaluated at 6 h, whereas other downstream assays were performed at 24 h. The experiments were conducted according to a previously described protocol [13].

### 2.3 Small Interfering RNA Transfection

To assess the involvement of the Nrf2 pathway, siRNA transfection was performed. HCECs at 80% confluence were transfected with either Nrf2‐targeting or scrambled control siRNAs using Lipofectamine 2000, following a brief complexation period in serum‐free medium. Cells were incubated for 24 h post‐transfection before use in downstream assays.

### 2.4 Measurements of Intracellular ROS

Intracellular oxidative stress levels were quantified using DCFH‐DA, a fluorescent ROS‐sensitive dye. After treatment, cells were incubated with the dye for 30 min and fluorescence was measured at 485/535 nm using a multi‐well plate reader.

### 2.5 Assessment of Nrf2 Transcriptional Activity and ELISA and Cytokine Profiling

Nuclear extracts were prepared from treated cells, and Nrf2 DNA‐binding activity was determined using a commercially available ELISA‐based TransAM assay. Signal detection was carried out using an antibody‐based colorimetric readout with tetramethylbenzidine substrate, and absorbance was recorded at 450 nm. Levels of antioxidant markers (HO‐1, GSH) and inflammatory cytokines were measured using commercially available ELISA kits and a multiplex cytokine antibody array. Analyses were performed in accordance with the respective manufacturers' protocols, and quantification was completed within one month post‐treatment.

## 3 Results and Discussion

**FIGURE 1:**
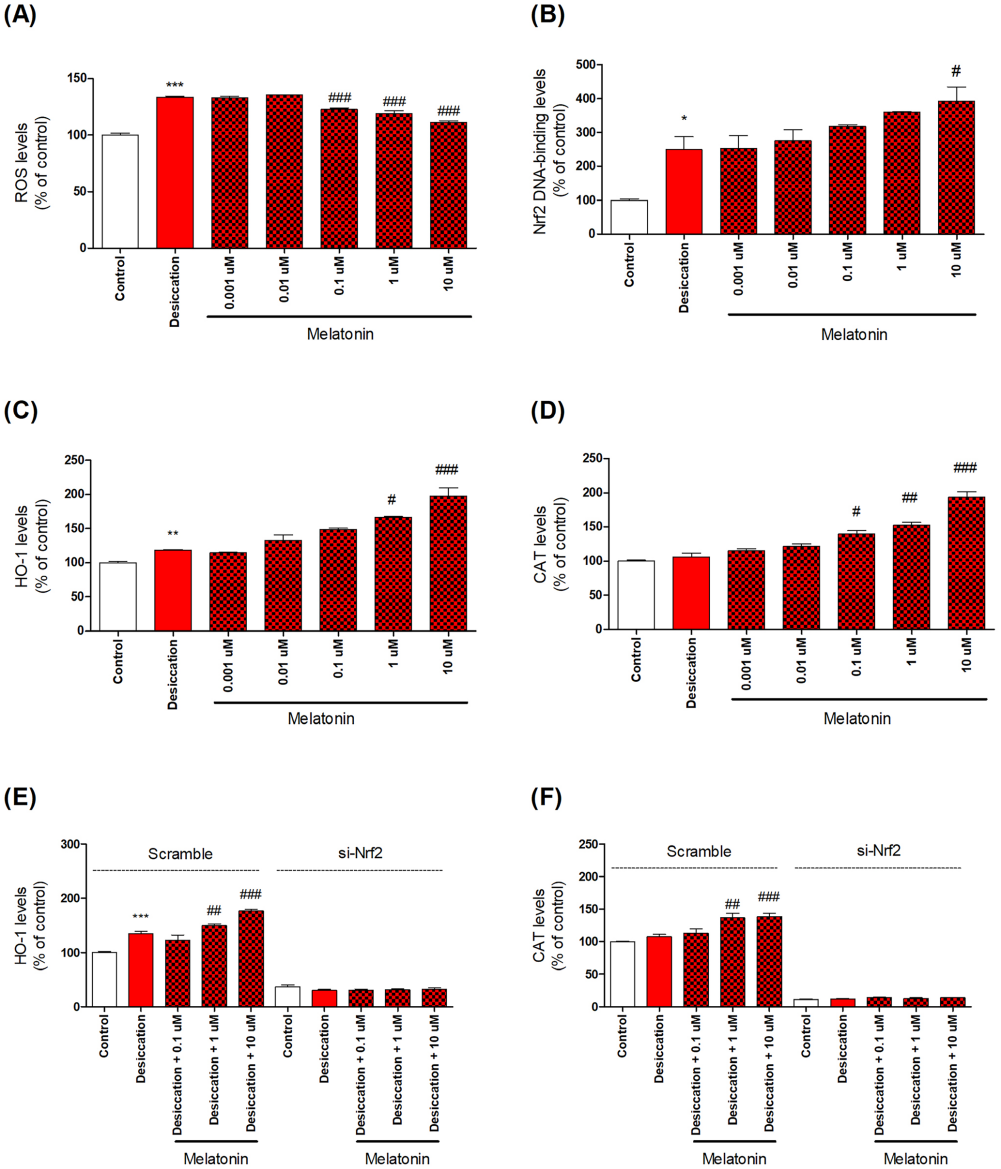
Melatonin treatment mitigates oxidative stress induced by desiccation in human corneal epithelial cells through activation of the Nrf2 signalling pathway. Intracellular ROS production was quantified (A), while ELISA assays were used to assess Nrf2 binding activity at 6 h of desiccation stress and the expression of downstream antioxidant enzymes at 24 h of desiccation stress, including HO‐1 and CAT (B–D). Panels E and F illustrate changes in antioxidant enzyme levels following melatonin exposure in both control and Nrf2‐silenced cells. Values are presented as the mean ± SEM. * *p* < 0.05, ** *p* < 0.01, and *** *p* < 0.001 versus the control; ^#^ *p* < 0.05, ^##^ *p* < 0.01 and ^###^ *p* < 0.001 versus the desiccation stress‐only group. Statistical analyses were performed using Prism version 7.0 (GraphPad Software, San Diego, CA, USA). Group differences were evaluated by one‐way ANOVA followed by Tukey's multiple comparison test, with *p* < 0.05 considered statistically significant.

**FIGURE 2:**
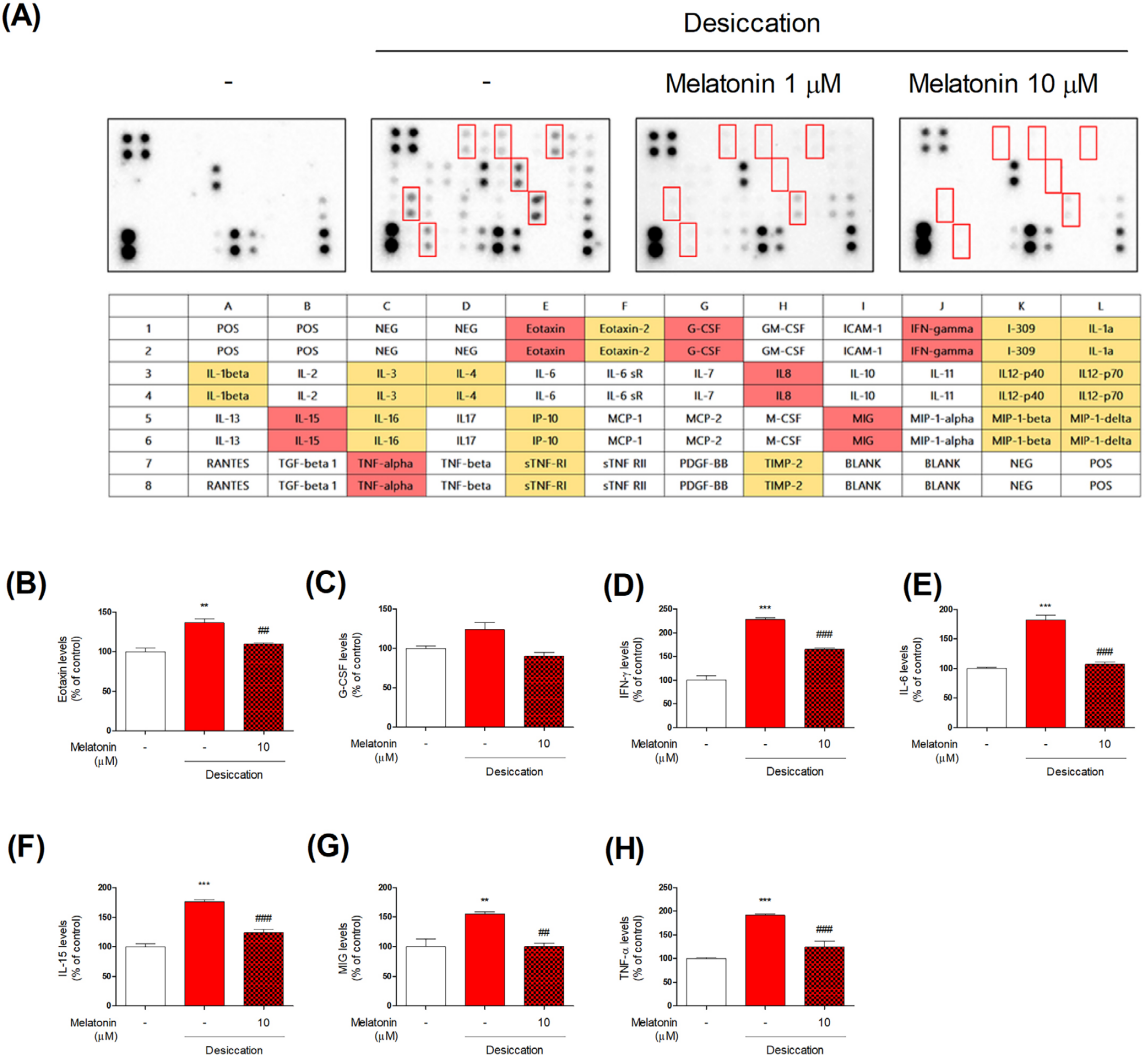
Effects of melatonin on desiccation stress‐induced inflammation cytokine signalling using antibody arrays. Densitometric ratios show differences in inflammatory cytokine expression. (A) Cytokine antibody arrays performed on HCECs exposed to desiccation stress with or without melatonin (1 and 10 μM) treatment. Red boxes highlight cytokines with markedly altered expression after melatonin treatment. The colour‐coded matrix represents expression changes: Red indicates significantly reduced expression, orange indicates moderate reductions, and yellow indicates mild changes. (B–H) Quantitative analysis of the cytokines Eotaxin (B), G‐CSF (C), IP‐10 (D), IL‐8 (E), IL‐15 (F), MIG (G) and TNF‐α (H) demonstrated that melatonin significantly downregulated their expression in a dose‐dependent manner. Values are expressed as the mean ± SEM. **p < 0.01 and ***p < 0.001 versus control; ^##^ *p* < 0.01 and ^###^ *p* < 0.001 vs. the desiccation‐stress only group. Statistical analyses were performed using Prism version 7.0 (GraphPad Software, San Diego, CA, USA). Group differences were evaluated by one‐way ANOVA followed by Tukey's multiple comparison test, with *p* < 0.05 considered statistically significant.

Melatonin has been increasingly studied as a pharmacological compound [5]. Several studies have reported the beneficial effects of melatonin on ocular health mediated through the suppression of oxidative stress [9, 12]. However, research on key antioxidant regulators such as Nrf2 remains insufficient. Herein, a network pharmacology analysis was conducted to predict the molecular mechanisms involved using TM‐MC 2.0. A total of 566 predicted targets of melatonin and 323 predicted targets related to ocular diseases were identified. Among them, 43 were common targets with a relevance score of 2 or higher, including TNF (7.32), CAT (5.21), HP (3.32), C3 (2.84), CASP3 (2.77), C2 (2.75), IL10 (2.69), RHO (2.29), and CRP (2.25). CAT is an enzyme that plays a crucial antioxidant role. In this study, we investigated the antioxidant effects and potential anti‐inflammatory properties of melatonin on DED (Figure 1). In the desiccation group, ROS levels increased to 133.49% ± 2.45%, but melatonin treatment suppressed this to 111.43% ± 1.69%. Additionally, CAT and HO‐1 expression were significantly elevated (approximately two‐fold) following melatonin treatment, indicating their role in enhancing antioxidant enzyme activity. Prior to these experiments, we performed an initial screening under H_2_O_2_‐induced oxidative stress conditions, in which Nrf2 activation and the upregulation of HO‐1 and CAT were clearly observed. These findings, presented in Figure S2, served as the basis for establishing melatonin's antioxidant potential and guided the subsequent analyses under desiccation stress. Antioxidants induce gene expression through Nrf2 [5]. Under normal conditions, Nrf2 binds to Kelch‐like ECH‐associated protein 1 (Keap1) in the cytoplasm [5, 13]. However, under oxidative stress, the thiol groups of Keap1 undergo oxidation, leading to the dissociation of Nrf2 [13]. Released Nrf2 translocates to the nucleus where it binds to the antioxidant response element (ARE) present in the promoters of various antioxidant enzymes and detoxifying protein genes [7, 13]. This induces the expression of CAT, GSH, and HO‐1. To confirm this, we assessed the DNA‐binding activity of Nrf2 and found that melatonin treatment enhanced its binding in a dose‐dependent manner [13]. To examine the effect of melatonin on the expression of antioxidant enzymes, we utilised Nrf2‐siRNA. The results indicated that, in the presence of Nrf2 knockdown, the effects of melatonin were diminished, suggesting that melatonin exerts its effects via the Nrf2 pathway. We assessed melatonin's anti‐inflammatory effects under desiccation stress using cytokine array (Figures 2 and S3). Desiccation upregulated inflammatory cytokines (e.g., TNF‐α, IL‐1β, IFN‐γ, IL‐8), consistent with oxidative stress–induced immune activation. Melatonin significantly reduced levels of Eotaxin, G‐CSF, IFN‐γ, IL‐8, IL‐15, MIG, and TNF‐α. Notably, TNF‐α and IL‐8 are known to impair epithelial barriers and drive chronic ocular inflammation [4, 14]. These findings, together with previous reports demonstrating that melatonin suppresses inflammation by reducing oxidative stress and activating Nrf2 signalling, provide stronger evidence that melatonin's anti‐inflammatory effects are mechanistically linked to its antioxidant properties [15]. Specifically, our results support that melatonin suppresses inflammatory responses in DED by attenuating oxidative stress–mediated cytokine cascades, consistent with the Nrf2‐dependent regulation of antioxidant defences [10]. Moreover, in Nrf2‐silenced HCECs, the inhibitory effect of melatonin on IL‐1ß expression was abolished, further confirming that its anti‐inflammatory action is at least partly mediated through Nrf2 signalling (Figure S4). Given that the antioxidant effects are Nrf2‐dependent, it is plausible that its anti‐inflammatory properties are partially mediated by the Nrf2 pathway. Thus, by targeting both oxidative stress and downstream inflammatory mediators, melatonin may play a dual role in modulating key pathogenic processes in DED. In this study, we primarily focused on demonstrating the antioxidant and anti‐inflammatory effects of melatonin under desiccation stress. However, some limitations should be noted. First, HO‐1 expression was upregulated even when Nrf2 binding activity did not markedly change, suggesting that HO‐1 may also be regulated through pathways independent of Nrf2. Second, while our data support a role for Nrf2 in mediating melatonin's effects, we did not perform an in‐depth mechanistic investigation of selective downstream targets of Nrf2. Further studies are warranted to clarify the precise contribution of Nrf2 and alternative signalling pathways to the observed responses.

In summary, our findings demonstrated that melatonin effectively mitigates oxidative stress and inflammation in a desiccation‐induced dry eye model. By activating the Nrf2 pathway, melatonin enhances the expression of key antioxidant enzymes and reduces ROS levels. Additionally, its ability to suppress pro‐inflammatory cytokine expression further supports its therapeutic potential. These results suggest that melatonin is a promising agent for treating DED by targeting oxidative stress and inflammatory pathways, thereby providing a novel strategy for ocular surface protection. Although this study demonstrated the antioxidant and anti‐inflammatory effects of melatonin in a desiccation‐induced in vitro model, in vivo validation using an animal model is essential for further translational applications.

## Author Contributions

**Hye‐Sun Lim:** methodology (equal), project administration (equal), resources (equal), validation (equal), visualization (equal), writing – original draft (equal). **Jinheung Park:** conceptualization (equal), methodology (equal), resources (equal), software (equal), writing – original draft (equal). **Woong‐Joo Whang:** formal analysis (equal), validation (equal), writing – original draft (equal). **Wan Seok Kang:** methodology (equal), visualization (equal), writing – original draft (equal). **Sunoh Kim:** validation (equal), writing – original draft (equal). **Young‐Sik Yoo:** funding acquisition (equal), investigation (equal), writing – original draft (equal). **Gunhyuk Park:** conceptualization (equal), formal analysis (equal), funding acquisition (equal), investigation (equal), supervision (equal), validation (equal), visualization (equal), writing – original draft (equal).

## Conflicts of Interest

The authors declare no conflicts of interest.

## Supporting information


**Figure S1:** jcmm70879‐sup‐0001‐FigureS1.docx.

## Data Availability

The datasets generated and/or analysed during the current study are available from the corresponding author upon reasonable request.
