# Supplementary material for: Melatonin Ameliorates Desiccation Stress‐Induced Ocular Inflammation in an In Vitro Model by Activating the Nrf2 Pathway
Source: J Cell Mol Med. 2025 Oct 16;29(20):e70879. doi: 10.1111/jcmm.70879 (PMC12530958; doi:10.1111/jcmm.70879)

**Supplemental figure 1.** Time-dependent changes in Nrf2 DNA-binding activity under desiccation stress. Human corneal epithelial cells were exposed to desiccation stress for the indicated time points (0, 1, 2, 6, 12, and 24 h). Nrf2 DNA-binding activity was measured using an ELISA-based assay and expressed as a percentage of the control (0 h). Bars represent mean ± SEM. A transient increase in Nrf2 activation was observed, peaking at 6 h and subsequently declining by 12–24 h. *p < 0.05, ***p < 0.001 vs. 0 h.


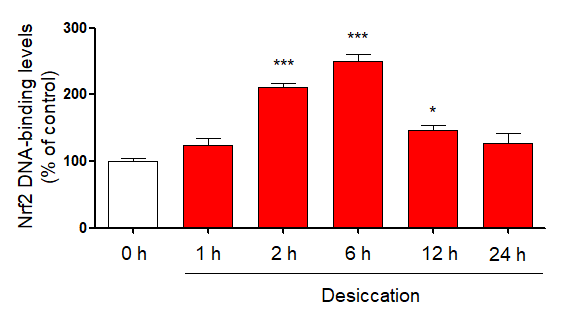


**Supplemental figure 2.** Effects of melatonin on Nrf2 activation and downstream antioxidant enzymes in H2O2-stimulated human corneal epithelial cells. Human corneal epithelial cells were exposed to H2O2 in the presence or absence of melatonin (5 μM). Nrf2 DNA-binding activity, HO-1 expression, and CAT expression were measured and expressed as a percentage of the control group. Bars represent mean ± SEM ***p < 0.001 vs. control; #p < 0.05, ###p < 0.001 vs. H₂O₂ alone. Melatonin treatment markedly enhanced Nrf2 DNA-binding activity and upregulated HO-1 and CAT expression compared with H₂O₂ treatment, indicating activation of the Nrf2 antioxidant pathway.


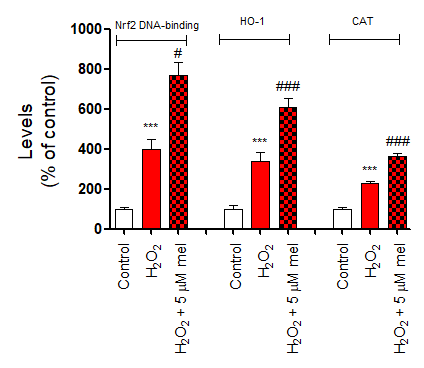


**Supplemental figure 3.** Effects of melatonin on desiccation stress-induced inflammation cytokine signaling using antibody arrays. Densitometric ratios show differences in inflammatory cytokine expression. (A) Bar graph showing the relative expression levels (% of control) of inflammatory cytokines under desiccation stress conditions.


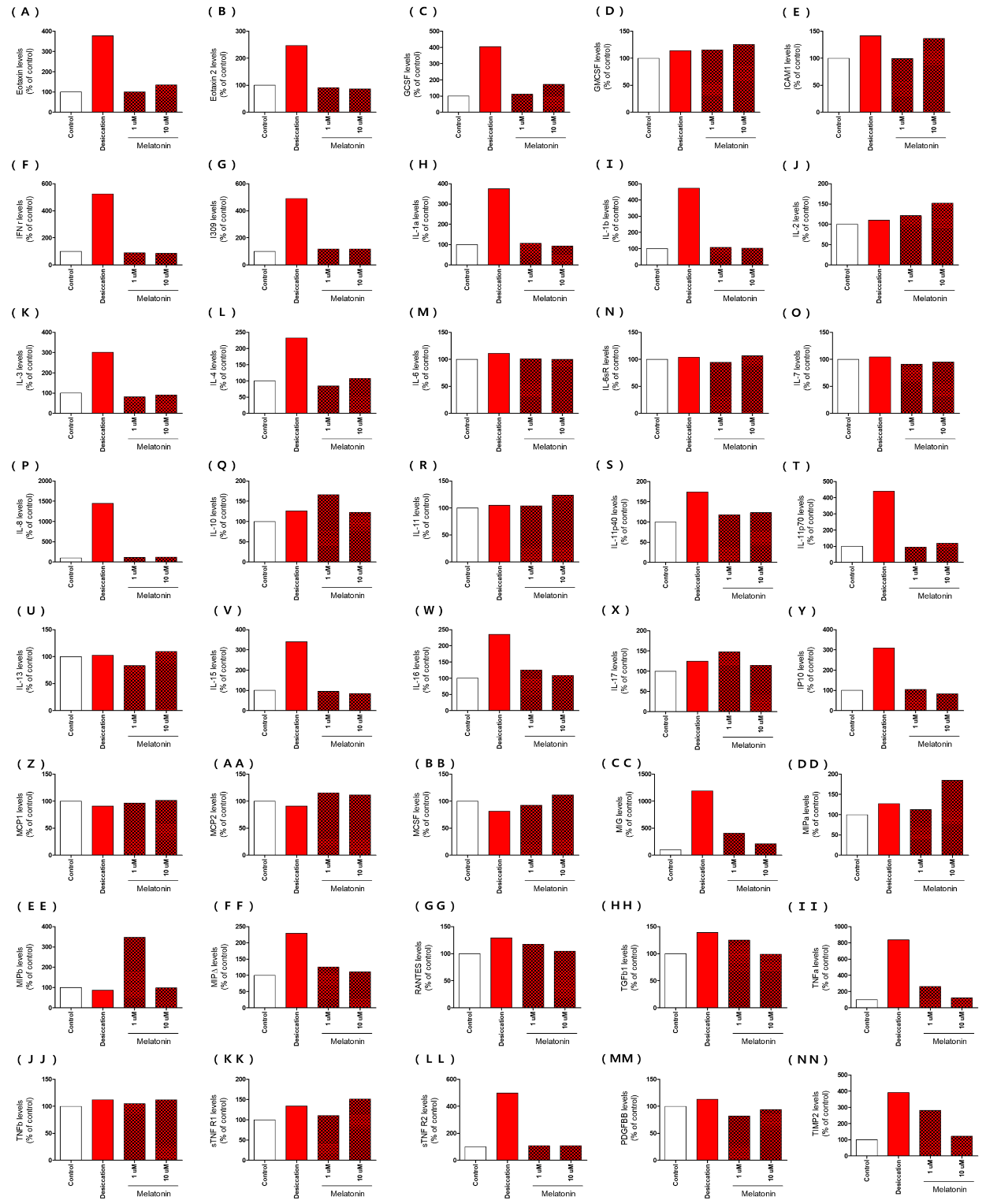


**Supplemental figure 4.** Effects of melatonin on IL-1ß expression in desiccation-stressed HCECs with or without Nrf2 silencing. Human corneal epithelial cells (HCECs) were transfected with either scramble siRNA or Nrf2-specific siRNA and then subjected to desiccation stress for 24 h in the presence or absence of melatonin (10 μM). IL-1ß protein levels were measured by ELISA and expressed as a percentage of the control. In scramble-transfected cells, desiccation markedly increased IL-1ß levels, which were significantly suppressed by melatonin treatment. However, this inhibitory effect of melatonin was abolished in Nrf2-silenced cells, indicating that melatonin’s anti-inflammatory action is at least partly dependent on Nrf2 signaling. Data are presented as mean ± SEM (n = X). *p < 0.05, ***p < 0.001 vs. control; ##p < 0.01 vs. desiccation alone.


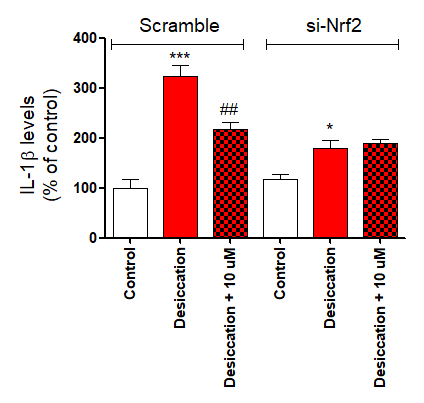

Supplement: Supplementary file 1 — Figure S1: jcmm70879‐sup‐0001‐FigureS1.docx. [file JCMM-29-e70879-s001.docx]
